# Multicenter Assessment of Radiation Exposure during Pediatric Cardiac Catheterizations Using a Novel Imaging System

**DOI:** 10.1155/2019/7639754

**Published:** 2019-10-31

**Authors:** Luke J. Lamers, Brian H. Morray, Alan Nugent, Michael Speidel, Petch Suntharos, Lourdes Prieto

**Affiliations:** ^1^Department of Pediatrics, Cardiology Division, University of Wisconsin School of Medicine and Public Health, Madison, WI 53792, USA; ^2^Department of Pediatrics, Cardiology Division, University of Washington School of Medicine, Seattle, WA 98195, USA; ^3^Department of Pediatrics, Cardiology Division, University of Texas Southwestern Medical Center, Dallas, TX 75235, USA; ^4^Department of Medical Physics, University of Wisconsin School of Medicine and Public Health, Madison, WI 53705, USA; ^5^Department of Pediatrics, Cardiology Division, The Cleveland Clinic, Cleveland, OH 44195, USA

## Abstract

**Objectives:**

To quantify radiation exposure during pediatric cardiac catheterizations performed by multiple operators on a new imaging platform, the Artis Q.zen (Siemens Healthcare, Forchheim, Germany), and to compare these data to contemporary benchmark values.

**Background:**

The Artis Q.zen has been shown to achieve significant radiation reduction during select types of pediatric cardiac catheterizations in small single-center studies. No large multicenter study exists quantifying patient dose exposure for a broad spectrum of procedures.

**Methods:**

Retrospective collection of Air Kerma (AK) and dose area product (DAP) for all pediatric cardiac catheterizations performed on this new imaging platform at four institutions over a two-year time period.

**Results:**

A total of 1,127 pediatric cardiac catheterizations were analyzed. Compared to dose data from earlier generation Artis Zee imaging systems, this study demonstrates 70–80% dose reduction (AK and DAP) for similar patient and procedure types. Compared to contemporary benchmark data for common interventional procedures, this study demonstrates an average percent reduction in AK and DAP from the lowest dose saving per intervention of 39% for AK and 27% for DAP for transcatheter pulmonary valve implantation up to 77% reduction in AK and 70% reduction in DAP for atrial septal defect closure.

**Conclusion:**

Use of next-generation imaging platforms for pediatric cardiac catheterizations can substantially decrease patient radiation exposure. This multicenter study defines new low-dose radiation measures achievable on a novel imaging system.

## 1. Introduction

Fluoroscopically guided diagnostic and interventional catheterizations play a vital role in the management of patients with congenital heart disease (CHD). Due to the increasing procedural complexity and frequent need for repeated studies, these cardiac catheterizations may account for more cumulative radiation exposure than all other imaging modalities combined throughout a CHD patient's lifetime. This radiation exposure often begins early in childhood, a time associated with greatest long-term risk for malignancies [1, 2]. With increased recognition of the radiation risks, there have been concerted efforts to decrease both patient and operator exposure through implementation of As Low As Reasonably Achievable (ALARA) principles and standards advocated by national radiation reduction quality improvement efforts [3, 4]. Through use of imaging guidelines set forth, several studies have documented significant reduction in radiation exposure during pediatric catheterizations [5–7], and the current trend is away from detailed high-quality imaging toward adequate image quality to perform procedures safely at the lowest acceptable dose.

While operators implement imaging techniques to lower dose, recent years have seen concerted efforts by manufacturers to reduce radiation exposure through technological processes. New system components and advanced image postprocessing algorithms offer potential for significant decrease in the radiation necessary for image generation [8–11]. Novel imaging systems are now available with complementary metal-oxide-semiconductor (CMOS) flat-panel x-ray detectors (FD) that increase the acquired image bit depth and employ crystalline silicon instead of amorphous silicon as a photodetector. Both improvements offer reduction in radiation dose to obtain similar image quality due to better digitalization and lower detector noise. In addition, x-ray tubes have been introduced that changed from classic coil filaments for photon generation to flat emitters, which maximize contrast, spatial, and temporal resolution through generation of more coherent focal spots, leading to further radiation dose reduction.

In light of ever-increasing complexity and duration of interventional procedures, the possibility of reducing radiation burden to both patient and user is of high interest. In this regard, a next-generation imaging platform, the Artis Q.zen, introduced by Siemens Healthcare (Forchheim, Germany) for commercial use in 2014 implements the above described FD and x-ray tube generation technologies and significantly decreases radiation doses while preserving image quality. Two small single-center studies assessing this imaging platform for pediatric cardiac catheterizations demonstrate 50–70% reduction in radiation exposure compared to previous generation Siemens imaging platforms [10] and to published benchmark data for a single intervention [11]. Variability in operator imaging strategies and system settings is known to exist between institutions, and a large patient population assessment of the radiation reduction capabilities of the Q.zen system does not exist. Thus, the primary aim of this study was to define radiation exposure for diagnostic and interventional catheterizations from a larger representative sample of pediatric CHD patients from multiple centers and to compare these exposure data to published contemporary benchmarks [12].

## 2. Methods

### 2.1. Data Collection

This study was conducted as a multicenter retrospective case review of radiation data for all consecutive pediatric cardiac catheterizations performed on Q.zen imaging platforms at four participating institutions from February 1, 2014 to September 1, 2016. IRB approval was obtained at each participating site in accordance with institutional requirements. Centers provided standard imaging protocols and system settings for comparison. CHD patients >18 years of age were excluded. Removal of the antiscatter grid and utilization of the air gap technique was standard imaging for patients <20 kg across centers. No comparative image analysis was performed.

All dose data reported were obtained from standard radiation summaries generated by the imaging system. The following variables were analyzed as measures of radiation exposure: Air Kerma (AK) expressed in units of mGy and dose area product (DAP) measured in units of *μ*G·m^2^. Fluoroscopy time, AK, and DAP data were recorded from each imaging plane and analyzed to reflect exposure attributed to fluoroscopy and cineangiography. In addition, the following procedural variables were recorded: patient age and weight and month/year of procedure to assess for trends in dose data over time. From these data, DAP per body weight (*μ*G·m^2^/kg) was calculated as a surrogate for energy delivered indexed by body weight. Although DAP/kg has been reported in previous studies and seems to be a valuable metric, it has never been validated as a method of reporting radiation exposure but was calculated for comparisons to published contemporary benchmarks [12].

For comparison to published pediatric studies [13], patients were stratified based upon procedure types and weight. Pooled data were analyzed for diagnostic catheterizations, right heart catheterization with biopsy, and interventional catheterizations for weights <5 kg, 5–12.5 kg, 12.5–25 kg, 25–45 kg, 45–65 kg, and >65 kg. In addition, radiation data from six isolated interventional procedures were identified and individually evaluated: (1) patent ductus arteriosus (PDA) closure, (2) atrial septal defect (ASD) closure, (3) pulmonary valvuloplasty, (4) aortic valvuloplasty, (5) coarctation intervention, and (6) transcatheter pulmonary valve placement (TPV) to facilitate comparison to contemporary studies [12].

Each participating site employed angiographic equipment with CMOS-based flat-panel detectors (Artis Q.zen, Siemens Healthineers, Forchheim, Germany) and was set up with custom imaging protocols that satisfied site-specific diagnostic and interventional needs in terms of image quality, impression, and frame rate. Each site used age discrimination for their acquisition protocols, accounting for the change in body composition from infant to young adult.

### 2.2. Statistical Analysis

The distributions of radiation exposure parameters were summarized in terms of median and interquartile ranges (IQR), stratified by weight groups, diagnosis, and intervention groups. Analysis of covariance (ANCOVA) was conducted to evaluate changes in total fluoroscopy time, cumulative Air Kerma, and cumulative DAP over time. Study center, weight, and age were included as covariates in this analysis. A linear trend test was conducted to evaluate whether there is a trend in total fluoroscopy time, cumulative Air Kerma, and cumulative DAP over time from the first quarter in 2014 to the third quarter in 2016. *P* < 0.05 was used to define statistical significance. Statistical analyses were conducted using SAS software (SAS Institute Inc., Cary, NC), version 9.4.

## 3. Results

### 3.1. Study Cohort and Imaging Protocols

Procedural and radiation data were available for 1,127 pediatric cardiac catheterizations from the four participating institutions. Basic patient demographics, case volumes, and procedural distributions by center are summarized in Table 1. Center B patients were older and weighed more than the cohort, and case distribution favored right heart catheterization with biopsy. Center D patients were younger and smaller with no isolated right heart catheterization data as this center does not have a pediatric heart transplant program.

Default weight-based detector dose rates, fluoroscopy, and cineangiography system settings are summarized in Tables 2 and 3. Pertinent differences between centers are observed for both operator imaging techniques and system settings. Center B imaging frame rates for fluoroscopy (4–7.5 pulses/s) and cineangiography (7.5–15 frames/s) were the lowest for all weights, and Center B system settings also had the lowest nGy/s for image generation for each weight classification.

### 3.2. Radiation Exposure by Procedure Type and Specific Intervention

Overall median fluoroscopy time was 15 minutes (IQR 8–27), median AK was 37 mGy (14–87), and median DAP was 224 *μ*Gy·m^2^ (84–671). The fraction of procedural AK from fluoroscopy was 45% (27–70%) with only a single case that exceeded 2,000 mGy. Measures of weight-based exposure data with interquartile ranges for diagnostic procedures, interventions, and right heart catheterizations with biopsy are summarized in Table 4. Comparing this cohort to reference data obtained on a previous generation imaging system from the same manufacturer (Artis Zee, Siemens Healthineers, Forchheim, Germany) [13] with similar fluoroscopy times and patient weights, the current study demonstrates a lowering in median AK from 135 to 37 mGy (73% reduction) and median DAP from 760 to 224 *μ*Gy·m^2^ (70% reduction), respectively. Similar dose reduction was demonstrated for diagnostic procedures (*n* = 312) (73% reduction in AK and 61% for DAP), interventions (*n* = 603) (79% reduction in AK and 75% for DAP), and right heart catheterizations (*n* = 214) (89% reduction in both AK and DAP).

Table 5 provides dose data for six selected interventional procedure types from the study cohort. Compared to contemporary radiation dose benchmarks from the prospective C3PO-QI study [12], the average percent reduction in AK and DAP ranged from the lowest dose saving per intervention of 39% for AK and 27% for DAP for transcatheter pulmonary valve (TPV) implantation up to 77% reduction in AK and 70% reduction in DAP for ASD closure. Percent reduction in DAP/kg values for the six individual interventions compared to the C3PO-QI was as follows: PDA closure 59% reduction, ASD closure 74% reduction, balloon aortic valve 66% reduction, balloon pulmonary valve 60% reduction, coarctation intervention 50% reduction, and TPV 23% reduction.

**Figure 1:**
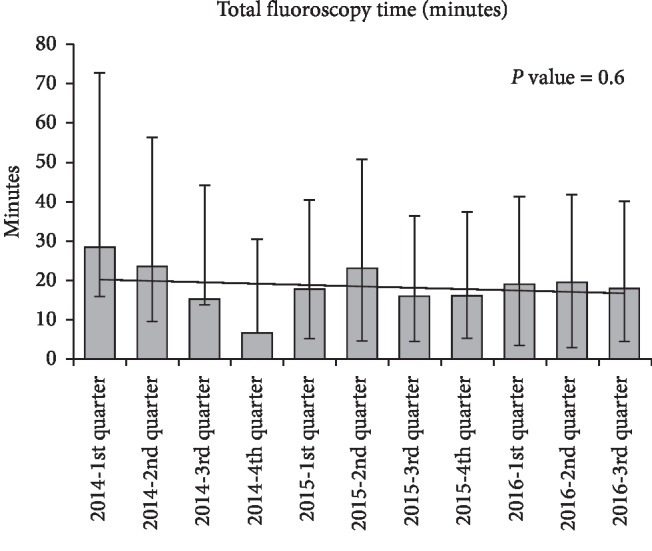
Quarterly total fluoroscopy time trends. *P* value = 0.6.

**Figure 2:**
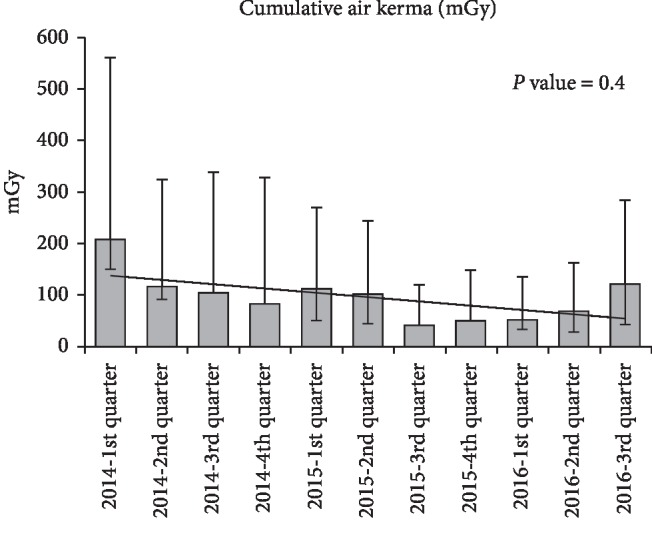
Quarterly cumulative Air Kerma trend across centers (adjusted by center, age, and weight). *P* value = 0.4.

**Figure 3:**
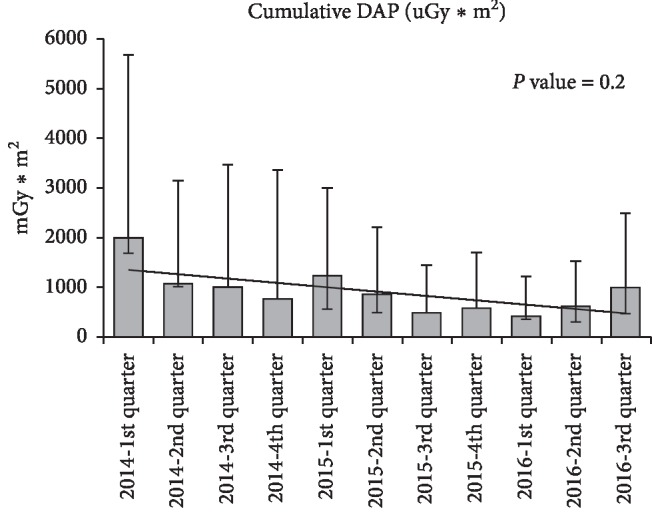
Quarterly cumulative dose area product (DAP) trend across centers (adjusted by center, age, and weight). *P* value = 0.2.

Figures 1–3 represent trends in quarterly procedural fluoroscopy time and dose (AK and DAP) for the cohort. There are no statistically significant trends in dose data over time. If the first quarter data of 2014 are eliminated, a timeframe during which the new system settings were being established, procedural dose trends are virtually unchanged from early 2014 through 2016, suggesting that the documented dose savings are attributed to system technical advances independent of operator practices.

## 4. Discussion

This is the first multicenter study of radiation exposure in pediatric CHD patients following cardiac catheterizations performed on a state-of-the art imaging system employing a new flat panel detector technology. This study demonstrates a large decrease in measured patient dose for diagnostic and interventional catheterizations when compared to data obtained from similar patients and procedures performed on a previous generation imaging system from the same manufacturer [13]. The present study also included a DAP/kg analysis for six selected interventional catheterization procedures for comparison to recently published data [12]. The DAP/kg data along with the standard measures total AK and DAP decreased substantially for all interventions.

As operators aim to decrease dose through application of ALARA strategies, decreasing fluoroscopy and cineangiography frame rates are simple and effective adjustments that can be made without significant compromise in image quality. Application of additional ALARA concepts such as limiting the number of cineangiograms to what is necessary, limiting use of lateral imaging, and avoiding unnecessary measurements that are available noninvasively has been shown to decrease radiation dose to less than we report [7, 14] without compromise to patient safety or procedural outcomes. It is well known that different pediatric centers use site-specific imaging protocols and system settings confirmed by the current study. The majority of imaging for this study was obtained at less than 10 pulses/second for fluoroscopy with one center consistently imaging at 4 pulses/second and less than 15 frames/second for cineangiograpy. Detector dose rates also varied by center and patient weights. Despite these variations in the participating center imaging techniques, we did not identify a center with consistently lower dose data when similar patient and procedure types were compared, and there was no trend toward lower dose over time with increased operator familiarity with the system. Thus, a major difference responsible for the reduced dose between the current study and that of Glatz et al. [13] appears to be due to the upgrade within the CMOS-based detectors of the system as kV limits are set to allow copper filtration at 0.2–0.6 mm and image postprocessing software for the two generations of equipment are similar. This technological advance generates lower noise within the detector for similar X-ray image impressions allowing a lower dose (15–23 nGy/pulse for the Q.zen vs. 23–29 nGy/pulse for the Artis Zee) to generate images.

The two main sources of fluoroscopic and cineangiography image degradation are quantum and electronic noise [15]. Quantum noise is caused by scattered photons due to interactions with objects in the x-ray beam. Electronic noise purely resides in the detector, and in contrast to quantum noise, does not vary with radiation dose. For conventional imaging, quantum noise dominates electronic noise as the limiting factor in image quality. In the past, dose settings have approached the limit at which electronic noise dominates quantum noise and no further dose reduction could occur. Since the introduction of flat-panel detectors, manufacturing material of the detector array has been amorphous silicon semiconductors, due to availability and ease of manufacturing. Only recently have detector-based CMOS elements become available that employ crystalline silicon instead of amorphous silicon as a photodetector for the interventional market. These CMOS-based detectors have the benefit of reduced electronic noise, with faster readout and less spatial blur [16]. This allows users to further reduce the dose per frame until a new, lower electronic noise threshold is met. This difference keeps the image quality and impression to the user constant, allowing use of the same image postprocessing, while lowering the patient dose.

The dose reduction documented in this study is largely due to technological advances in detector properties. Other manufactures have recently achieved similar degrees of dose reduction between system generations with technologic advances within other parts of the image generation pathway. Sullivan et al. report radiation data pre- and postupgrade of an AlluraXper FD 20/10 system to the AlluraClarity (Philips Healthcare, Best, the Netherlands) and demonstrate use of Clarity was associated with a 58% reduction in DAP for all pediatric cardiac catheterization procedures after adjustment for fluoroscopy time, BSA, and procedure type, albeit from a high level of radiation exposure with the prior system generation [8]. AlluraClarity is a software upgrade to the fluoroscopy system that digitally enhances images obtained with lower radiation doses through technological advances within the image acquisition chain. The system image postprocessing software and hardware were upgraded, while the x-ray tube, the biplane flat-panel detectors, and other image acquisition equipment was not, so the dose savings were largely due to image postprocessing as there was no change in image generation, filtration, or collimation capabilities [8]. Similar dose reduction has been demonstrated for adults with use of Clarity technology for coronary angiography and angioplasty and vascular and neurovascular interventions [17–19]. Ideally, manufacturers will continue to scrutinize each step in the image generation process searching for further dose saving, image preserving technologies, while at the same time researchers experiment with alternative interventional imaging strategies such as MRI-guided procedures that would eliminate procedural radiation exposure.

Efforts to reduce lifetime radiation exposure are important, particularly in CHD patients who are often exposed to high-lifetime doses as a result of repeated procedures and diagnostic testing. Every pediatric radiation exposure study discusses the theoretical long-term increase risk for malignancy that is estimated to be 6.5% greater than baseline for CHD patients with the highest levels of radiation exposure [1]. Operator's diligent application of ALARA concepts has the greatest potential to decrease radiation exposure [7, 14]. In addition, this study demonstrates that use of upgraded imaging systems can reduce radiation exposure by 25–75%, depending on procedure type compared to published radiation dose benchmarks in pediatric cardiac catheterization [12]. This information should prompt children's hospitals to consider modernizing their imaging systems to significantly reduce exposure to all who work and require procedures within pediatric catheterization laboratories.

## 5. Study Limitations

Image quality was not directly evaluated in this study or in the previous publications which served as a basis of comparison. Consequently, it was not possible to determine if the differences in dose were correlated with differences in image quality. Furthermore, there were variations in patient population, procedure type, and operator experience across the participating sites, which were difficult to control for. A study design that controls for these factors may enable more precise comparisons of dose and determination of the dose reduction attributable to the x-ray technology.

## Figures and Tables

**Table 1:** Center-specific case data.

Center	Age (months) Median (range)	Weight (kg) Median (range)	Diagnostic (*n*)	Intervention (*n*)	RHC ± biopsy (*n*)
A	41 (0–215)	13.5 (2.4–97)	128	229	76
B	88 (0–216)	23 (2.4–85)	25	51	81
C	42 (0–214)	14 (2.2–131)	89	188	56
D	14 (0–215)	9.2 (1.1–81)	69	135	0
Cohort	37 (0–216)	13.2 (1.1–131)	311	603	213

**Table 2:** Fluoroscopy detector dose rate by center and patient weight.

Center	Weight								Average of all categories
	<6 kg		<20 kg		<40 kg		≥40 kg		
	Pulse/s	nGy/s	Pulse/s	nGy/s	Pulse/s	nGy/s	Pulse/s	nGy/s	nGy/s
A	7.5	135	7.5	135	10	180	7.5	217.5	166.9
B	7.5	75	7.5	172.5	4	92	4	92	107.9
C	7.5	135	7.5	195	7.5	217.5	7.5	270	204.4
D	10	150	10	150	7.5	172.5	7.5	172.5	161.3

**Table 3:** Cineangiography detector dose rate by center and patient weight.

Center	Weight								Average of all categories
	<6 kg		<20 kg		<40 kg		≥40 kg		
	Frame/s	nGy/s	Frame/s	nGy/s	Frame/s	nGy/s	Frame/s	nGy/s	nGy/s
A	15	1800	15	1800	15	2100	10	1400	1775
B	15	1800	7.5	900	7.5	900	7.5	900	1125
C	15	1800	15	1800	15	2100	15	2100	1950
D	30	2300	15	1500	15	1800	15	2100	1950

Note: detector dose rate (nGy/s) is the product of frame rate (frame/s) and programmed dose per frame (nGy/frame).

**Table 4:** Study cohort median radiation exposure parameters and interquartile range (IQR) stratified by weight for diagnostic procedures, interventions, and RHC with biopsy.

	Weight (kg)	*N*	Air Kerma (mGy) from both planes		DAP (*μ*Gy m^2^) from both planes		DAP/kg (*μ*Gy m^2^/kg) from both planes		Fluoroscopy time (min) from both planes		Fraction Air Kerma from fluoroscopy	
			Median	IQR	Median	IQR	Median	IQR	Median	IQR	Median	IQR
Diagnostic	0–5	42	22	12–45	93	55–174	25	13–43	21	9–31	0.41	0.24–0.50
	5–12.5	119	25	14–53	137	76–307	22	11–47	18	12–26	0.37	0.24–0.53
	12.5–25	94	32	19–67	266	151–612	16	9–37	14	8–21	0.33	0.21–0.51
	25–45	22	75	37–129	682	450–1173	21	13–37	12	6–28	0.48	0.19–0.70
	45–65	17	110	69–227	1326	721–3017	22	13–57	12	8–16	0.34	0.17–0.48
	>65	18	133	28–382	1565	432–4540	19	6–45	7	3–19	0.43	0.24–0.87

Intervention	0–5	123	32	17–60	120	53–198	33	16–61	21	12–40	0.48	0.32–0.69
	5–12.5	208	35	18–75	174	90–496	24	12–63	22	12–39	0.42	0.26–0.57
	12.5–25	130	42	19–82	278	131–678	18	8–37	19	11–30	0.47	0.33–0.64
	25–45	70	100	46–386	1081	469–2535	34	15–77	20	10–30	0.46	0.27–0.72
	45–65	41	199	104–659	2689	1244–7353	53	21–151	20	11–32	0.53	0.35–0.85
	>65	31	206	71–968	3522	843–6978	46	9–90	15	9–21	0.49	0.34–0.67

RHC biopsy	5–12.5	30	6	1–25	38	5–158	4.0	1–13	7	4–14	0.95	0.30–1.00
	12.5–25	73	23	1–60	157	12–373	9	1–21	9	5–14	0.51	0.28–1.00
	25–45	34	8	1–92	88	14–751	2	0–27	7	4–10	0.94	0.32–1.00
	45–65	46	8	2–136	91	37–1577	2	1–28	6	4–9	1.00	0.37–1.00
	>65	31	12	3–327	177	39–530	3	1–58	6	3–14	1.00	0.57–1.00

**Table 5:** Study cohort median radiation exposure parameters and interquartile range (IQR) stratified by intervention type.

Intervention	*N*	Air Kerma (mGy) from both planes		DAP (*μ*Gy m^2^) from both planes		DAP/kg (*μ*Gy m^2^/kg) from both planes		Fluoroscopy time (min) from both planes		Fraction Air Kerma from fluoroscopy	
		Median	IQR	Median	IQR	Median	IQR	Median	IQR	Median	IQR
ASD closure	58	24	12–83	229	93–990	9.0	5–23	12	8–19	0.8	0.61–0.96
PDA closure	109	27	17–45	135	75–253	15	9–24	13	9–19	0.5	0.35–0.59
Balloon dilation pulmonary valve	45	29	11–47	116	47–326	21	11–31	16	10–21	0.4	0.2–0.52
Balloon dilation aortic valve	25	65	33–152	394	136–1601	34	17–53	15	12–28	0.4	0.15–0.46
Coarctation intervention	46	101	34–386	598	176–2649	45	22–90	19	11–27	0.4	0.2–0.44
Transcatheter pulmonary valve	17	889	539–1425	9869	6850–16616	197	153–249	51	30–62	0.5	0.26–0.68

## Data Availability

The data used to support the findings of this study are included within the article.

## Abbreviations

CHD: Congenital heart disease
ALARA: As low as reasonably achievable
CMOS: Complementary metal-oxide-semiconductor
FD: Flat-panel X-ray detectors
AK: Air kerma
mGy: Milligray
DAP: Dose area product
*μ*G·m^2^: microgray × meter squared
*μ*G·m^2^/kg: Dose area product divided by kilograms
PDA: Patent ductus arteriosus
ASD: Atrial septal defect
TPV: Transcatheter pulmonary valve.
